# The GLP-1 Analogue Exenatide Improves Hepatic and Muscle Insulin Sensitivity in Diabetic Rats: Tracer Studies in the Basal State and during Hyperinsulinemic-Euglycemic Clamp

**DOI:** 10.1155/2014/524517

**Published:** 2014-11-16

**Authors:** Hui Wu, Chunhua Sui, Hui Xu, Fangzhen Xia, Hualing Zhai, Huixin Zhang, Pan Weng, Bing Han, Sichun Du, Yingli Lu

**Affiliations:** Institute and Department of Endocrinology and Metabolism, Shanghai Ninth People's Hospital Affiliated Shanghai Jiaotong University School of Medicine, Shanghai 200011, China

## Abstract

*Objective*. Glucagon-like peptide-1 (GLP-1) analogues (e.g., exenatide) increase insulin secretion in diabetes but less is known about their effects on glucose production or insulin-stimulated glucose uptake in peripheral tissues.* Methods*. Four groups of Sprague-Dawley rats were studied: nondiabetic (control, C); nondiabetic + exenatide (C + E); diabetic (D); diabetic + exenatide (D + E) with diabetes induced by streptozotocin and high fat diet. Infusion of 3-^3^H-glucose and U-^13^C-glycerol was used to measure basal rates of appearance (R_a_) of glucose and glycerol and gluconeogenesis from glycerol (GNG). During hyperinsulinemic-euglycemic clamp, glucose uptake into gastrocnemius muscles was measured with 2-deoxy-D-^14^C-glucose.* Results*. In the diabetic rats, exenatide reduced the basal R_a_ of glucose (*P* < 0.01) and glycerol (*P* < 0.01) and GNG (*P* < 0.001). During the clamp, R_a_ of glucose was also reduced, whereas the rate of disappearance of glucose increased and there was increased glucose uptake into muscle (*P* < 0.01) during the clamp. In the nondiabetic rats, exenatide had no effect.* Conclusion*. In addition to its known effects on insulin secretion, administration of the GLP-1 analogue, exenatide, is associated with increased inhibition of gluconeogenesis and improved glucose uptake into muscle in diabetic rats, implying improved hepatic and peripheral insulin sensitivity.

## 1. Introduction

Diabetes is characterized by high blood glucose, which is caused by a variety of mechanisms: increased gluconeogenesis, decreased glucose uptake, and insulin resistance (IR) in liver and skeletal muscle which are the most important factors.

Glucagon-like peptide-1 (GLP-1) is a gut-derived incretin hormone which regulates glucose homeostasis. It has a range of important effects on islet cells, promoting insulin gene transcription and beta-cell proliferation and differentiation. GLP-1 inhibits glucagon secretion through *α* and *δ* cells in the islet [1, 2]. It also contributes to delay gastric emptying and satiety promotion [3]. However, the potential effect of GLP-1 on the regulation of glucose metabolism requires further investigations.

Exenatide is a GLP-1 analogue which is naturally resistant to the proteolytic activity of DPP-IV and has a long-term, highly specific role as a GLP-1 agonist [4] to reduce blood glucose levels in diabetic patients [5]. But, there is a lack of studies concerning the mechanism of effect of GLP-1 analogues on gluconeogenesis (GNG), hepatic glucose production (HGP), or glucose disappearance (Rd) and glucose uptake in gastrocnemius muscle in dynamic and steady-state situations. Furthermore, most of these are limited in vitro studies or clinical observational studies [6, 7].

In the current study, we used isotope tracer technology to dynamically explore the effect of GLP-1 analogues on hepatic and extrahepatic glucose homeostasis in diabetic rats.

## 2. Materials and Methods

### 2.1. Animals

The Sprague-Dawley rats (6 weeks, male, 130 g) were established diabetic model according to a published protocol [8]. The diabetic rats were fed a high-fat diet (40% of calories from fat) for 10 weeks, followed by a single low-dose intraperitoneal injection of streptozotocin (STZ, 30 mg/kg, Sigma, St. Louis, MO, USA), whose plasma glucose level increased to 16.7 mmol/L within 3 days after STZ treatment and which remained at this level throughout the study period. The nondiabetic control rats were fed a chow diet (10.3% of calories from fat) and received an intraperitoneal injection of citrate-phosphate buffer of the same volume as that given to the diabetic model rats. Nondiabetic rats were assigned either to treatment with vehicle (nondiabetic control, C, *n* = 6) or with exenatide (nondiabetic + exenatide, C + E, *n* = 6). Diabetic rats were also assigned to one of two treatments: vehicle-treated (diabetic, D, *n* = 5) or exenatide treated (diabetic + exenatide, D + E, *n* = 5). All animal procedures conformed to the ethical principles in animal research adopted by the Department of Laboratory Animal Science, Jiaotong University School of Medicine, Shanghai, China.

### 2.2. Treatment of Exenatide

Intervention with exenatide (Eli Lilly, USA) was initiated on the third day after STZ injection and lasted for 8 weeks. Exenatide was administered by subcutaneous injection at a dose of 5 *μ*g/day. The control group rats were given the same volume of normal saline solution every day.

### 2.3. Isotope Infusion and Measurement

**Figure 1:**
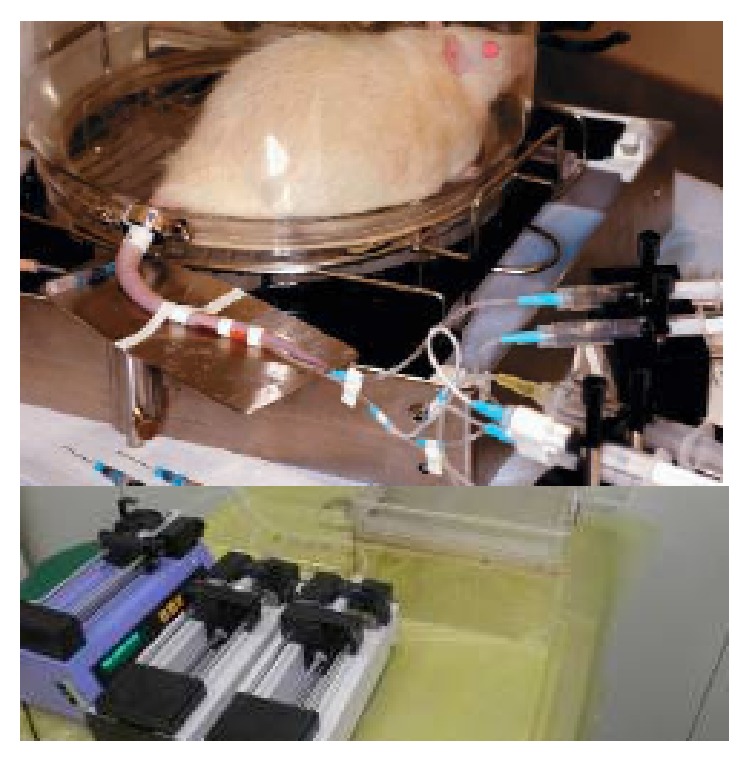
Tracer perfusion platform in the rat. Catheters were inserted into the tail artery and vein. This procedure used small incisions and enabled the rats to be kept conscious and relaxed throughout the experiment. It was constantly infused through the i.v. infusion line driven by a Harvard mini-infusion pump (Harvard Apparatus, Holliston, MA, USA) for 75 min (basal period). The infusion rates were 0.5 *μ*Ci/kg/min (3-^3^H-glucose) and 0.84 *μ*mol/kg/min (U-^13^C-glycerol). Blood was sampled prior to infusion of tracer and at 65, 70, and 75 min. Subsequently, while the 3-^3^H-glucose infusion continued, a primed and continuous infusion of recombinant human insulin 5 mIu/kg/min was initiated for another 90 min. The plasma glucose concentration was kept constant at the basal level by monitoring the plasma glucose every 10 minutes and empirically adjusting the infusion rate of a 50% glucose solution. Twenty minutes before the end of the chase period, 1 *μ*Ci of 2-deoxy-D-^14^C-glucose was injected through the i.v. infusion line to enable measurement of tissue glucose uptake. During the final 10 minutes, 3 more blood samples were collected at 5-minute intervals.

**Figure 2:**
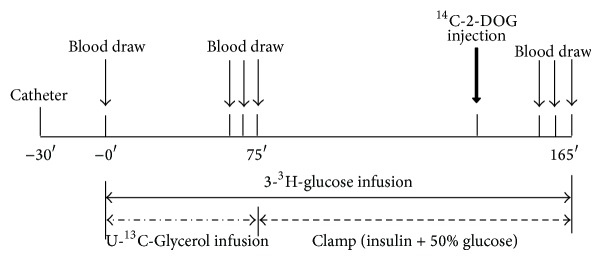
Schematic diagram of the tracer infusion protocol. The process of blood collection and tracer infusion from beginning to end.

All rats were fasted overnight (12–14 hours) and studied the following morning. After local anesthesia with lidocaine, catheters were inserted into the lateral tail vein for infusion of tracers and in the tail artery for blood sampling as described previously [9]. Throughout the experiments, animals were conscious and relaxed; they groomed normally and drank water periodically or sat calmly, indicating that stress resulting from the procedures was minimal (Figure 1). 3-^3^H-Glucose (PerkinElmer, Waltham, MA, USA) and U-^13^C-glycerol (Cambridge Isotope, Andover, MA, USA) were constantly infused through the i.v. infusion line driven by a Harvard mini-infusion pump (Harvard Apparatus, Holliston, MA, USA) for 75 min (basal period). The infusion rates were 0.5 *μ*Ci/kg/min (3-^3^H-glucose) and 0.84 *μ*mol/kg/min (U-^13^C-glycerol). Blood was sampledprior to infusion of tracer and at 65, 70, and 75 min. Subsequently, while the 3-^3^H-glucose infusion continued, a primed and continuous infusion of recombinant human insulin (5 mIu/kg/min, Novolin R, Novo Nordisk, Denmark) was initiated for another 90 min (chase period). The plasma glucose concentration was kept constant at the basal level by monitoring the plasma glucose every 10 minutes and empirically adjusting the infusion rate of a 50% glucose solution. Twenty minutes before the end of the chase period, 1 *μ*Ci of 2-deoxy-D-^14^C-glucose (PerkinElmer) was injected through the i.v. infusion line to enable measurement of tissue glucose uptake. During the final 10 minutes, 3 more blood samples were collected at 5-minute intervals. A flow chart of the study design is shown in Figure 2. The rats were euthanized by heart opening under anesthesia with pentobarbital (50 mg/kg) to reduce the blood content of the tissues. A biopsy was promptly taken from gastrocnemius muscle, cut into small pieces and immersed in liquid nitrogen, and stored at −80°C. Plasma samples were prepared on ice, centrifuged at 4°C, separated, and stored at −80°C until assayed.

Glucose concentration was determined by the glucose oxidase method (Beckman Glucose Analyzer, Beckman instruments, Fullerton, CA, USA). Insulin concentration was measured using a magnetic affinity immunoassay (Insulin MPAIA Kit). Plasma lipid concentrations were assayed using a Siemens Dimension MAX integrated chemistry system (Siemens Healthcare Diagnostics Inc.).

An acid digestion method was applied to blood samples and gastrocnemius tissue before determining ^3^H and ^14^C radioactivity by liquid scintillation counting (LS6500 Multipurpose Scintillation Counter, Beckman, USA) as previously described [10]. Enrichment of glycerol and glucose was measured by GC-MS analysis of their trimethylsilyl derivatives. Briefly, 1 *μ*1 of the samples was injected (splitless mode) into a gas chromatograph (Auto System XL GC, PerkinElmer) equipped with a 30 m fused silica capillary column (DB-5MS, Agilent, USA) and interfaced with a mass spectrometer (TurboMass MS, PerkinElmer) operating in the electronic impact ionization mode. The carrier gas was helium. The operating conditions were as follows: injector at 270°C and oven at 70°C for 4 min and then increased to 300°C at 10°C/min. Ions with mass-to-charge ratios (*m/z*) of 218 (unlabeled glycerol) and 221 (labeled glycerol) were selectively monitored. The peak area ratio 221/218 was calculated, and the corresponding enrichment (MPE) was determined from standard curves containing weighted amounts of natural and U-^13^C-glycerol and injected before and after biological samples. Similarly, ions with mass-to-charge ratios (*m/z*) of 319 (unlabeled glucose) and 322 (labeled 1,2,3-^13^C-glucose) were also selectively monitored. The peak area ratio 322/319 was calculated, and the corresponding enrichment was determined.

### 2.4. Calculations

Turnover rates (R_a_) of glucose and glycerol were calculated with the steady-state equation from the respective tracer infusion rates (*F*) and enrichment (expressed as specific activity (glucose) or MPE (glycerol)). Glucose R_aglu_ was calculated from the 3-^3^H-glucose data, while glycerol R_agly_ was determined from the U-^13^C-glycerol data. The detailed formulae were as follows [11].

Calculation of gastrointestinal glucose absorption is as follows:

(1) Ragluμmol/kg/min⁡ =HGP =Fgluμci/kg/min⁡plasma  3-H3-glucose  specific  activity  μci/μmol.

Calculation of gastrointestinal glycerol absorption is as follows:

(2) Raglyμmol/kg/min⁡ =Fglyμmol/kg/min⁡plasma  U-C13-glycerol  MPE−Fglyμmol/kg/min⁡.

The gluconeogenesis (GNG) from glycerol was calculated from the ^13^C enrichment of glucose and glycerol as follows [12].

Calculation of glycerol gluconeogenesis rates is as follows:

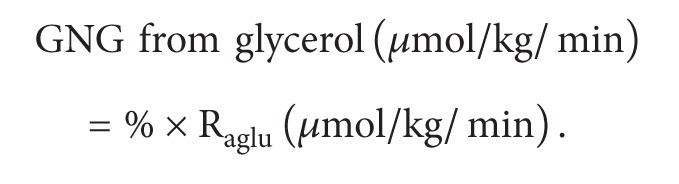
(3)
Calculation of the percent glycerol converted to glucose is as follows:

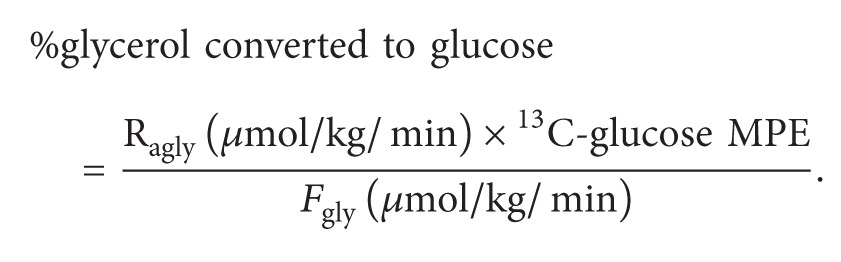
(4)
The MPE of glucose and glycerol was calculated by curve fitting the enrichment with an exponential fit and extrapolating to the plateau of enrichment.

In the basal state, hepatic glucose production (HGP) is equal to the glucose R_a_ after an overnight fast. When blood glucose is in a constant state, R_aglu_ = disappearance rate of glucose (R_dglu_), while, during the hyperinsulinemic clamp studies, HGP is equal to the difference between the total glucose R_aglu_ determined by tracer analysis and the directly measured glucose infusion rate (GIR) which helps to maintain euglycemia. The average insulin-stimulated glucose uptake rate of gastrocnemius muscle in the steady state was calculated as previously described [13].

Calculation of glucose intake per unit mass of skeletal muscle is as follows:

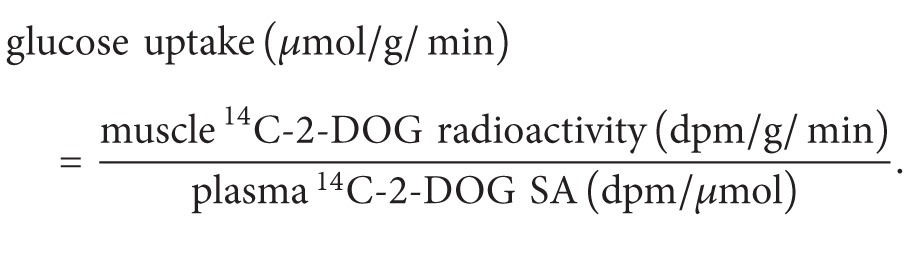
(5)


### 2.5. Statistical Analysis

The statistical package for the social sciences (SPSS version 17.0) was used for data analysis. All results are expressed as mean ± SD and values were compared using one-way ANOVA followed by the Student-Newman-Keuls* post hoc* test for multiple comparisons as appropriate. Differences were defined as significant at *P* < 0.05.

## 3. Results

### 3.1. Body Weight and Fasting Blood Glucose

There were no significant differences in body weight or fasting blood glucose levels between the four group rats before the experiment. However, a significant reduction in body weight was seen in both D + E and C + E groups (Table 1).

The fasting blood glucose in the D + E group decreased compared with the D group. It also decreased in the C + E group rats compared with those in the C group (*P* < 0.05) (Table 1).

### 3.2. Fasting Plasma Insulin (FINS) Levels, Homeostasis Model-Insulin Resistance (HOMA-IR), and Insulin Sensitivity Index (ISI)

The FINS concentration in the D + E group (18.86 ± 2.48 *μ*Iu/mL) was significantly lower than that in the D group rats (29.09 ± 3.61 *μ*Iu/mL,  *P* < 0.01), while no difference was found between C group rats (12.84 ± 5.26 *μ*Iu/mL) and the C + E group rats (15.00 ± 5.55 *μ*Iu/mL). The HOMA-IR in the D + E group rats (7.54 ± 1.58) was also significantly lower than that in the D group rats (18.40 ± 3.84,  *P* < 0.01), while it was still significantly higher than that in the C group rats (3.19 ± 1.38,  *P* < 0.01) and the C + E group (2.79 ± 0.94,  *P* < 0.01). Although the HOMA-IR in the C + E group rats was lower than that in the C group rats, this was not significant (Table 1).

The reverse pattern of ISI was observed with the D + E group rats having a higher insulin sensitivity index (−5.12 ± 0.21) than the D group rats (−6.01 ± 0.21), although this was still significantly lower than the C group rats (−4.15 ± 0.61, *P* < 0.01) and the C + E group rats (−4.09 ± 0.35,  *P* < 0.01). The C + E group rats had a higher insulin sensitivity index than the C group rats, but this was not significant (Table 1).

### 3.3. Plasma Lipid Profile

The D + E group rats had lower plasma total cholesterol, triglycerides, and low density lipoprotein-cholesterol level (1.61 ± 0.11 mmol/L, 0.44 ± 0.07 mmol/L, and 0.43 ± 0.13 mmol/L, resp.) than the D group rats (1.85 ± 0.16 mmol/L, 0.63 ± 0.08 mmol/L, and 0.61 ± 0.11 mmol/L, resp.); this was still significantly higher than the C group rats (1.19 ± 0.23 mmol/L, 0.12 ± 0.04 mmol/L, and 0.23 ± 0.04 mmol/L, resp.) and the C + E group rats (1.02 ± 0.14 mmol/L, 0.19 ± 0.06 mmol/L, and 0.20 ± 0.05 mmol/L, resp.). There was no significant difference in the plasma lipid spectrum between the rats in the C group and those in the C + E group (Table 1).

### 3.4. Basal Rate of Appearance of Glucose

Basal insulin concentrations were as follows: C, 12.84 ± 5.26 *μ*Iu/mL; C + E, 15.00 ± 5.55 *μ*Iu/mL; D, 29.09 ± 3.61 *μ*Iu/mL; and D + E, 18.86 ± 2.48 *μ*Iu/mL. Corresponding rates of appearance of glucose were as follows: C, 42.27 ± 10.56 *μ*mol/kg/min; C + E, 35.11 ± 3.96 *μ*mol/kg/min; D, 121.07 ± 16.55 *μ*mol/kg/min; and D + E, 94.70 ± 13.46 *μ*mol/kg/min. The D + E group rats had lower glucose appearance rates than the D group rats (*P* < 0.01), while it had still significantly higher than the C group rats (*P* < 0.01) and the C + E group rats (*P* < 0.01). The C + E group rats also had a lower glucose appearance rates than the C group rats, but no significant difference was found (Figure 5(b)).

### 3.5. Basal Glycerol Appearance and Gluconeogenesis from Glycerol (GNG)

**Figure 3:**
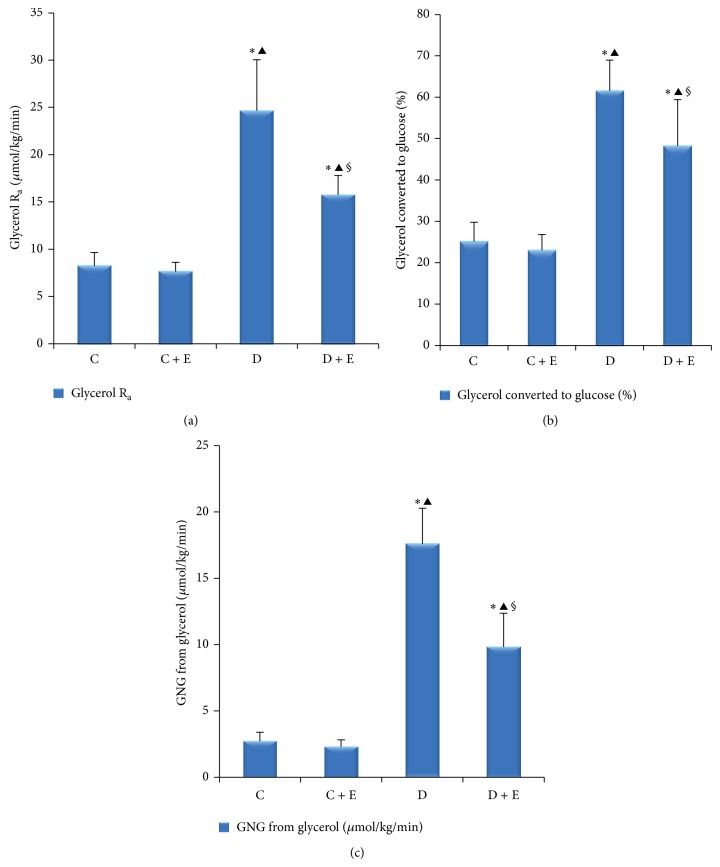
Plasma parameters of glycerol flux and gluconeogenesis. C: nondiabetic, control; C + E: nondiabetic + exenatide; D: diabetic; D + E: diabetic + exenatide. R_a_ means appearance rate; GNG means gluconeogenesis. Data are presented as mean ± SD. ^*^ *P* < 0.01; ^#^ *P* < 0.05 compared with C group. ^▲^ *P* < 0.01 compared with C + E group. ^**§**^ *P* < 0.01 compared with D group. Glycerol appearance after an overnight fast was lower (*P* < 0.01) in the D + E group rats than that in the D group rats (a). The gluconeogenesis from glycerol, such as glycerol converted to glucose (%) and gluconeogenesis from glycerol, was clearly lower (*P* < 0.01) in the D + E group rats than that in the D group rats. (b and c). ^*^ *P* < 0.01, ^#^ *P* < 0.05 compared with basal. ^*β*^ *P* < 0.01, ^*α*^ *P* < 0.05 compared with C group. ^*σ*^ *P* < 0.01, ^*γ*^ *P* < 0.05 compared with C + E group. ^**§**^ *P* < 0.01,  ^*φ*^ *P* < 0.05 compared with D group.

The glycerol appearance after an overnight fast was lower (*P* < 0.01) in the D + E group rats than that in the D group rats (15.75 ± 2.04 versus 24.65 ± 5.39 *μ*mol/kg/min). The gluconeogenesis from glycerol was all considerably lower (*P* < 0.01) in the D + E group rats than that in the D group rats [glycerol converted to glucose (%): 48.23 ± 11.20% versus 61.53 ± 7.44%; gluconeogenesis from glycerol: 9.76 ± 2.54 *μ*mol/kg/min versus 17.54 ± 2.68 *μ*mol/kg/min] (Figure 3).

### 3.6. Clamp Concentrations of Blood Glucose and Insulin

**Figure 4:**
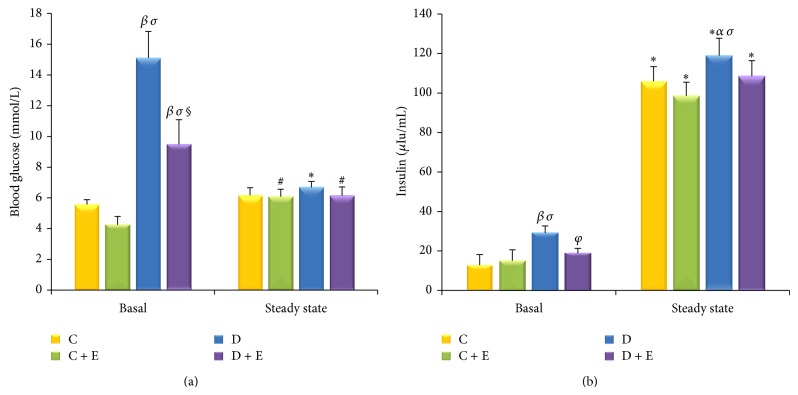
Glucose and insulin concentration during the hyperinsulinemic-euglycemic clamp steady state. The concentrations of blood glucose during the clamp steady state were similar among the four groups of rats. (a) Plasma insulin concentrations of the rats were dramatically elevated compared with their basal state (*P* < 0.01). Furthermore, plasma insulin concentrations in the D group rats were significantly higher than those in the C group rats (*P* < 0.05) and in the C + E group rats (*P* < 0.01) under the clamp steady-state conditions (b). ^*^ *P* < 0.01, ^#^ *P* < 0.05 compared with basal. ^*β*^ *P* < 0.01, ^*α*^ *P* < 0.05 compared with C group. ^*σ*^ *P* < 0.01, ^*γ*^ *P* < 0.05 compared with C + E group. ^**§**^ *P* < 0.01,  ^*φ*^ *P* < 0.05 compared with D group.

After the exogenous insulin infusion (5 mIu/kg/min) for 90 minutes, the concentrations of blood glucose at clamp steady state were similar among the four group rats. At the same time, the plasma insulin concentrations of the rats were dramatically elevated compared with their basal state (*P* < 0.01). Specifically, the plasma insulin concentrations of the C group rats increased to 105.95 ± 7.38 *μ*Iu/mL (the steady state), of the C + E group rats increased to 98.60 ± 6.86 *μ*Iu/mL, of the D group rats increased to 118.92 ± 8.81 *μ*Iu/mL, and of the D + E group rats increased to 108.47 ± 7.91 *μ*Iu/mL. Meanwhile, under the clamp steady state, the plasma insulin concentrations in the D group rats were also significantly higher than those in the C group rats (*P* < 0.05) and those in the C + E group rats (*P* < 0.01) (Figures 4(a) and 4(b)).

### 3.7. Clamp Glucose Infusion Rates

The exogenous glucose infusion rates required to maintain the glucose levels at the clamp point (6-7 mmol/L) were higher (*P* < 0.01) in the D + E group rats than those in the D group rats (144.68 ± 11.03 versus 114.50 ± 9.40 *μ*mol/kg/min), while they were still significantly lower (*P* < 0.01) than those in the C group rats (144.68 ± 11.03 versus 178.71 ± 8.73 *μ*mol/kg/min). The glucose infusion rates did not differ between the C group rats and the C + E group rats (178.71 ± 8.73 versus 177.95 ± 9.89 *μ*mol/kg/min) (Figure 6(a)).

### 3.8. Clamp Rate of Appearance of Glucose

**Figure 5:**
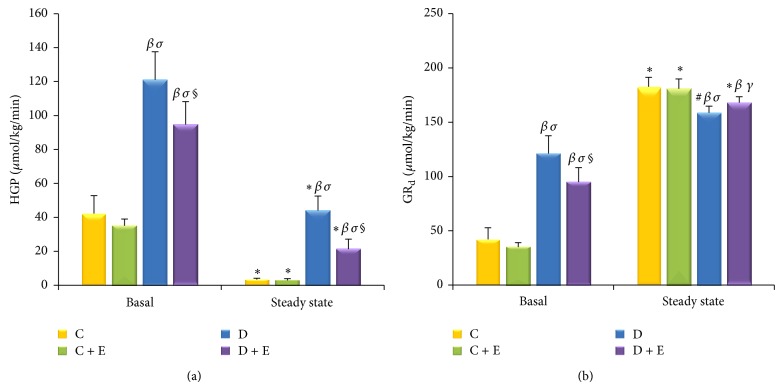
HGP and the disappearance rate of glucose (GR_d_) during the hyperinsulinemic-euglycemic clamp steady state. HGP following an overnight fast was higher (*P* < 0.01) in the D group rats than that in the D + E group rats. During clamp steady state, HGP was greater (*P* < 0.01) in the D group rats than that in the D + E group rats (a). In the basal state, the D + E group rats had a lower GR_d_ than the D group rats (*P* < 0.01). The C + E group rats also had a lower GR_d_ than the C group rats, but this was not significantly different (b). Under the clamp steady state, the GR_d_ of the D group rats was lower than that of the C group rats (*P* < 0.05) and that of the C + E group rats (*P* < 0.01). The GR_d_ of the D + E group rats was also significantly lower than that of the C group rats (*P* < 0.01) and that of the C + E group rats (*P* < 0.05). There was no significant difference found between the C group rats and the C + E group rats (*P* > 0.05) (b). ^*^ *P* < 0.01, ^#^ *P* < 0.05 compared with basal. ^*β*^ *P* < 0.01, ^*α*^ *P* < 0.05 compared with C group. ^*σ*^ *P* < 0.01, ^*γ*^ *P* < 0.05 compared with C + E group. ^**§**^ *P* < 0.01,  ^*φ*^ *P* < 0.05 compared with D group.

Under the euglycaemic, hyperinsulinaemic conditions of the clamp, R_a_ of glucose was greater (*P* < 0.01) in the D group rats than that in the D + E group rats (44.14 ± 8.49 *μ*mol/kg/min versus 21.37 ± 5.89 *μ*mol/kg/min) (Figure 5(a)).

### 3.9. Clamp Glucose Disappearance Rates

After exogenous insulin infusion (5 mIu/kg/min) for 90 minutes, the glucose disappearance rates of the C group rats increased from 42.27 ± 10.56 to 182.16 ± 9.15 *μ*mol/kg/min (*P* < 0.01), and increment of 139.9 *μ*mol/kg/min, of the C + E group rats increased from 35.11 ± 3.96 to 180.87 ± 8.85 *μ*mol/kg/min (*P* < 0.01), an increment of 145.76 *μ*mol/kg/min, of the D group rats increased from 121.07 ± 16.55 to 158.63 ± 6.06 *μ*mol/kg/min (*P* < 0.05), an increment of 37.56 *μ*mol/kg/min, and of the D + E group rats increased from 94.70 ± 13.46 to 167.92 ± 5.50 *μ*mol/kg/min (*P* < 0.01), an increment of 73.22 *μ*mol/kg/min.

Under the clamp steady state, although the plasma insulin concentrations of the D group rats were higher than those of the C group rats (*P* < 0.05) and the C + E group rats (*P* < 0.01), their glucose disappearance rates were still lower than those of the C group rats (*P* < 0.05) and the C + E group rats (*P* < 0.01). At the same time, the glucose disappearance rates of the D + E group rats were significantly lower than those of the C group rats (*P* < 0.01) and the C + E group rats (*P* < 0.05). Although the glucose disappearance rates of the D + E group rats were higher than those of the D group rats, this was not significant (*P* > 0.05). There was also no significant difference found between the C group rats and the C + E group rats (*P* > 0.05) (Figure 5(b)).

### 3.10. Glucose Uptake in Muscle

**Figure 6:**
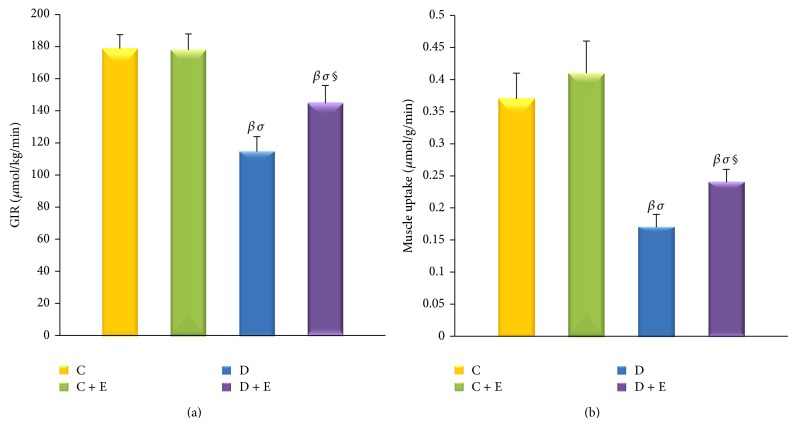
Exogenous glucose infusion rates (GIR) and 2-deoxyglucose uptake into muscle. The GIR required to maintain the glucose levels at the clamp point (6-7 mmol/L) was higher (*P* < 0.01) in the D + E group rats than that in the D group rats, while it was still significantly lower (*P* < 0.01) than that in the C group rats. The GIR did not differ between the C group rats and in the C + E group rats (a). Exenatide treatment for 8 weeks markedly increased glucose uptake in diabetic rat gastrocnemius compared with the vehicle-treated diabetic rats (*P* < 0.01). Increased glucose uptake was also found in the C + E group rats compared with the C group rats, but this did not reach statistical significance (b). C: nondiabetic control; C + E: nondiabetic + exenatide; D: diabetic; D + E: diabetic + exenatide. Data are presented as mean ± SD. ^*^ *P* < 0.01, ^#^ *P* < 0.05 compared with basal. ^*β*^ *P* < 0.01, ^*α*^ *P* < 0.05 compared with C group. ^*σ*^ *P* < 0.01, ^*γ*^ *P* < 0.05 compared with C + E group. ^**§**^ *P* < 0.01,  ^*φ*^ *P* < 0.05 compared with D group.

Exenatide treatment for 8 weeks markedly increased glucose uptake in diabetic rat gastrocnemius compared with that of vehicle-treated diabetic rats (0.24 ± 0.02 versus 0.17 ± 0.02 *μ*mol/g/min, *P* < 0.01). Increased glucose uptake was also found in the C + E group rats compared with the C group rats but this was not statistically significant (0.41 ± 0.05 versus 0.37 ± 0.04 *μ*mol/g/min) (Figure 6(b)).

## 4. Discussion 

Tracer techniques are widely used in research of the metabolism of glucose and of other molecules. Previous tracer studies in rat models have often involved invasive surgical placement of catheters in the carotid artery and jugular vein [14, 15]. Such procedures often require large, deep incisions and extension of catheters through the subcutaneous space-up to the dorsal neck area, which obviously cause stress and may, therefore, require 4–7 days of recovery after surgery. Frequent clotting in these catheters is another problem. All of the above conditions could considerably interfere with the metabolic flux of the substrate and, thus, affect the results.

In the current study, we used a novel approach, in which the catheters were inserted into the rats' tail artery and vein. This procedure required only small incisions and thus rats could be kept conscious and relaxed throughout the experiment. This led to a low level of stress throughout the experiment, thus avoiding disturbance to glucose and lipid metabolism (Figure 1).

Many gastrointestinal hormones, such as GLP-1, orexin, neuropeptide Y, and motilin, play an important role in body's metabolism through regulation of food intake and energy consumption [16]. GLP-1 analogues have a similar effect to other gastrointestinal hormones [17].

In our study, weight loss was observed in both diabetic and nondiabetic rats treated with exenatide, consistent with the appetite reducing effects of GLP-1 analogues and confirming that exenatide affects the body weight of both diabetic and healthy individuals. Fasting blood glucose, insulin, and HOMA-IR were significantly decreased and ISI increased, and there was an accompanying reduction in cholesterol, triglycerides, and low density lipoprotein-cholesterol level.

The results showed glucose and glycerol appearance rates in exenatide-treated diabetic rats were much lower than those in the nontreated diabetic rats. Peripheral adipose tissue degradation is the main source of glycerol during fasting. Because of the lack of glycerokinase in adipose tissue, the glycerol released by lipolysis cannot be resynthesized into adipose tissue, and therefore glycerol appearance rates in the blood are also a reliable indicator for lipolysis [18]. Therefore, it appears that lipolysis in the diabetic rats was partially inhibited by exenatide.

Reductions in basal gluconeogenesis and lipolysis could result from increased insulin secretion, but fasting insulin concentrations were reduced in exenatide-treated rats. The alternative possibility is that exenatide increased sensitivity to insulin. As described above, HOMA-IR was significantly decreased and ISI increased, consistent with improvements in insulin sensitivity and with previous observations of the effects of exenatide on insulin sensitivity [19, 20]. Previous work on normoglycemic dogs has found improved insulin sensitivity with exenatide, but there appears to be no work on effects of exenatide on insulin sensitivity in a diabetic animal model [21]. There are limitations on the precision of measuring insulin sensitivity through determination of HOMA and ISI but, in the present study, the technique of isotope tracer provides more dynamic data on metabolic flux of carbohydrate with measurement of hepatic glucose production and peripheral glucose uptake to directly quantify insulin sensitivity.

Hepatic insulin resistance is frequently seen, even in the prediabetic state [22]. Gluconeogenesis is one of the major sources of fasting endogenous glucose production (EGP), which maintains the body's fasting blood glucose within normal levels [23, 24]. However, the hepatic insulin resistance seen in diabetes is a major cause of the high level of gluconeogenesis in diabetes.

We used U-^13^C-glycerol as a tracer to monitor the naturally occurring kinetics of glycerol metabolism in the different rat groups. U-^13^C-Glycerol takes exactly the same chemical structure and function as natural glycerol in the blood, except that three ^12^C atoms in the carbon chain are replaced by ^13^C. It will follow the same metabolic pathway and not disturb the endogenous glycerol metabolism. Thus, it precisely reflects endogenous glycerol metabolism. These sets of data showed that gluconeogenesis declined significantly (including glycerol converted to glucose (%) and gluconeogenesis from glycerol) in the exenatide-treated diabetic rats compared with the vehicle-treated diabetic rats. This means that exenatide has the ability to decrease gluconeogenesis in diabetes.

The hyperinsulinemic-euglycemic clamp was used in association with 3-^3^H-glucose infusion. Under hyperinsulinemic conditions, HGP in control rats in the steady-state conditions was almost completely suppressed (more than 90%) by insulin in contrast to the continuing hepatic glucose production in the diabetic group. Although hepatic insulin resistance was still found in the exenatide-treated diabetic rats, their HGP under the clamp steady-state had been reduced by more than 50% compared with the vehicle-treated diabetic rats. Thus, these data provide further evidence that exenatide can reduce hepatic insulin resistance in diabetic rats.

During these experiments, the disappearance rates of glucose in the control rats under hyperinsulinemia increased more than 300 percent. In the exenatide-treated diabetic rats a 70 percent increase in glucose disappearance rate was observed, but, in vehicle-treated diabetic rats, there was only a 19 percent increase in glucose disappearance. Accordingly, to maintain the clamp glucose level, the exogenous glucose infusion rates needed to be increased by almost 28 percent in the exenatide compared with the vehicle-treated diabetic rats. These results clearly show that exenatide enhances the effect of insulin on glucose uptake.

Skeletal muscle insulin resistance is the primary defect in diabetes [25], preceding the failure of beta cell function and overt hyperglycemia [26, 27]. Approximately 80–90% of exogenous glucose infusion is taken up by skeletal muscle [28, 29]. 2-Deoxy-glucose has many of the same features as normal glucose; it is able to be taken up by tissues and phosphorylated to form 6-P-glucose in the cells. However, no further metabolism occurs, and it is a classic method for measuring glucose uptake ability in target tissues [13]. We used 2-deoxy-D-1-^14^C-glucose to determine the average glucose uptake rates of skeletal muscle under the clamp steady state.

The glucose uptake rates of gastrocnemius were lowest in the nontreated diabetic rats, with glucose uptake being 46 percent of the uptake rate in the normal control rats. The average glucose uptake rates of gastrocnemius in exenatide-treated diabetic rats were approximately 65 percent of the uptake in the nondiabetic control rats and were 41 percent higher than in the vehicle-treated diabetic rats. This demonstrated that the skeletal muscle insulin resistance was improved by exenatide.

In conclusion, the glucagon-like peptide-1 analog, exenatide, does significantly inhibit hepatic gluconeogenesis and increases glucose uptake in the peripheral muscle tissues to improve hepatic and extrahepatic insulin resistance in diabetic rats.

## Figures and Tables

**Table 1:** Characteristics of rats in the four groups at the end of study.

	C (*n* = 6)	C + E (*n* = 6)	D (*n* = 5)	D + E (*n* = 5)
Body weight (g)	531.9 ± 37.6	474.3 ± 24.9^*^	468.2 ± 29.0^*^	397.6 ± 13.7^∗▲*φ*^
FBG (mmol/L)	5.57 ± 0.31	4.25 ± 0.55^#^	15.10 ± 1.73^∗▲^	9.48 ± 1.61^∗▲§^
FINS (*μ*Iu/mL)	12.84 ± 5.26	15.00 ± 5.55	29.09 ± 3.61^∗▲^	18.86 ± 2.48^*φ*^
HOMA-IR	3.19 ± 1.38	2.79 ± 0.94	18.40 ± 3.84^∗▲^	7.54 ± 1.58^∗▲§^
ISI	−4.15 ± 0.61	−4.09 ± 0.35	−6.01 ± 0.21^∗▲^	−5.12 ± 0.21^∗▲§^
TC (mmol/L)	1.19 ± 0.23	1.02 ± 0.14	1.85 ± 0.16^∗▲^	1.61 ± 0.11^∗▲*φ*^
TG (mmol/L)	0.12 ± 0.04	0.19 ± 0.06	0.63 ± 0.08^∗▲^	0.44 ± 0.07^∗▲§^
LDL (mmol/L)	0.23 ± 0.04	0.20 ± 0.05	0.61 ± 0.11^∗▲^	0.43 ± 0.13^∗▲§^

C: nondiabetic control; C + E: nondiabetic + exenatide; D: diabetic; D + E, diabetic + exenatide.

FBG: fasting blood glucose; FINS: fasting plasma insulin; ISI: insulin sensitivity index.

Data are presented as mean ± SD.

^*^ *P* < 0.01, ^#^ *P* < 0.05 compared with normal rats (C).

^▲^ *P* < 0.01 compared with normal rats treated with exenatide (C + E).

^§^ *P* < 0.01, ^*φ*^ *P* < 0.05 compared with diabetic rats treated with vehicle (D).
